# Risk of hip fracture among older people using antihypertensive drugs: a nationwide cohort study

**DOI:** 10.1186/s12877-015-0154-5

**Published:** 2015-12-01

**Authors:** Sabine Ruths, Marit S. Bakken, Anette H. Ranhoff, Steinar Hunskaar, Lars B. Engesæter, Anders Engeland

**Affiliations:** Department of Global Public Health and Primary Care, University of Bergen, PO Box 7804, N-5020 Bergen, Norway; Research Unit for General Practice, Uni Research Health, Bergen, Norway; Kavli Research Centre for Geriatrics and Dementia, Haraldsplass Deaconess Hospital, Bergen, Norway; Department of Clinical Science, University of Bergen, Bergen, Norway; National Centre for Emergency Primary Health Care, Uni Research Health, Bergen, Norway; Department of Clinical Medicine, University of Bergen, Bergen, Norway; Norwegian Arthroplasty Registry, Department of Orthopaedics, Haukeland University Hospital, Bergen, Norway; Department of Pharmacoepidemiology, Norwegian Institute of Public Health, Oslo, Norway

**Keywords:** Older people, Hip fracture, Antihypertensive drugs, Bone mineral density, Fall

## Abstract

**Background:**

Many people with a high risk of hip fracture have coexisting cardiovascular diseases. We aimed to examine associations between exposure to antihypertensive drugs and the risk of hip fracture among older people.

**Methods:**

We conducted a cohort study of the 906,422 people born before 1945 and living in Norway in 2005. We obtained information on all prescriptions of antihypertensive drugs dispensed (the Norwegian Prescription Database) in 2004–2010 and the dates of primary hip fractures (the Norwegian Hip Fracture Registry) in 2005–2010. We compared the incidence rates of hip fracture during the time people were exposed and unexposed to antihypertensive drugs by calculating the standardized incidence ratio (SIR).

**Results:**

Altogether, 39,938 people experienced a primary hip fracture (4.4 %). The risk of hip fracture was decreased among people exposed to thiazides (SIR 0.7, 95 % confidence interval (CI) 0.6–0.7), beta-blockers (SIR 0.7, 95 % CI 0.7–0.8), calcium channel blockers (SIR 0.8, 95 % CI 0.8–0.8), angiotensin II receptor blockers (SIR 0.8, 95 % CI 0.7–0.8), ACE inhibitor/thiazide combination products (SIR 0.7, 95 % CI 0.6–0.7) and angiotensin II receptor blocker/thiazide combination products (SIR 0.6, 95 % CI 0.6–0.6). Use of loop diuretics and ACE inhibitors (plain products) was associated with increased fracture risk in people born after 1924, and with decreased risk in those born before 1925. The protective associations were stronger among exposed men than among exposed women for all drugs except loop diuretics. The SIRs decreased with increasing age among exposed people, except for thiazides and angiotensin II receptor blockers.

**Conclusions:**

We found a reduced risk of hip fracture associated with overall use of most antihypertensive drugs, but an increased risk with loop diuretics and ACE inhibitors among people younger than 80 years and in new users of loop diuretics. This may have great impact at the population level, because the use of antihypertensive drugs is widespread in people at risk of hip fracture. Clinical studies are needed to further explore these associations.

**Electronic supplementary material:**

The online version of this article (doi:10.1186/s12877-015-0154-5) contains supplementary material, which is available to authorized users.

## Background

Hip fractures represent critical events that seriously affect morbidity and mortality [1]. The risk of hip fracture increases with age; with estimated lifetime risk of 30 % for women and 18 % for men in Norway at the age of 50 years [2]. Many older people with osteoporosis have coexisting cardiovascular disease such as hypertension, ischemic heart disease, heart failure and stroke. Both high blood pressure and systolic hypotension have been associated with falls, reduced bone mineral density (BMD) and hip fracture [3–6].

Antihypertensive drugs are prescribed to more than half the people aged 60 years and older in Europe and the United States, in accordance with recommendations of broad risk assessments, lower limits for blood pressure and ambitious therapeutic goals [7–9]. While observational studies indicate an increased risk of falls and hip fracture in older people after initiating antihypertensive drugs [10–12], meta-analyses have revealed no associations between long-term treatment and falls [13, 14]. A review of pharmacology, mechanisms and possible effects on BMD revealed beneficial effects of thiazides (reduced renal calcium depletion, direct stimulation of osteoblasts), beta-blockers (inhibition of beta-adrenergic receptor in bone), and angiotensin-converting enzyme (ACE) inhibitors (inhibition of ACE in local renin-angiotensin-aldosterone system); no effect in either direction of angiotensin II receptor blockers (direct blockade of angiotensin II receptor) and calcium channel blockers (inhibition of voltage-gated calcium channel); negative effects of loop diuretics (increased renal calcium depletion, risk of falls) [15].

Previous studies of associations between antihypertensive drug use and hip fracture have focused mainly on diuretics and beta-blockers. While research involving renin-angiotensin-aldosterone active agents and calcium channel blockers is limited and diverging, combination products have not yet been considered. Because these drugs are widely used, it is important to compare their impact with other antihypertensive drug groups to inform prescribing decisions for older people at risk of osteoporosis and hip fracture. Based on the Norwegian Prescription Database and the Norwegian Hip Fracture Registry, we conducted a nationwide cohort study to examine associations between exposure to antihypertensive drugs (plain and combination products) and the risk of hip fracture among people 60 years and older.

## Methods

We conducted a cohort study based on data extracted from three national registries: the Norwegian Prescription Database (NorPD) [7], the Norwegian Hip Fracture Registry [16] and the Central Population Registry [17]. Registries with clinical data on comorbidity were not available. The model has previously been described in detail [18].

### Registries

The NorPD is a central health registry that contains information on all dispensed prescriptions at pharmacies to individual patients treated in ambulatory care in Norway since January 2004 [19]. For the purpose of this study, we extracted data on all prescriptions of antihypertensive drugs (defined below), dispensed from 1 January 2004 until 31 December 2010, by the items’ Anatomical Therapeutic Chemical (ATC) system code and drug volume by defined daily dose (DDD) to individual patients [20]. Prescriptions dispensed during 2004 were used to identify current drug users when the study period started on 1 January 2005. The NorPD does not capture individual information on drugs dispensed to people staying in hospitals or nursing homes (about 12,000 and 40,000 at any time). Further, the NorPD does not comprise clinical data. Reimbursement codes linked to diagnoses became available from 2009; because they were not validated against clinical data, we chose not to include this information.

Since January 2005, all hospitals in Norway performing operations for hip fracture have reported information on such surgery to the Norwegian Hip Fracture Registry [16]. The data extracted for this study comprised the date of first hip fracture during the study period (hereafter referred to as primary hip fracture), or the date of surgery in case of missing information, for the period from 1 January 2005 until 31 December 2010. Although the registry also contains fractures among patients in nursing homes and hospitals, these individuals cannot be identified in the study cohort.

The Central Population Registry contains demographic information on the entire population of Norway since 1960 [17]. We extracted data on the year of birth, sex and the date of death or emigration if applicable.

The variables selected from these three registries were merged, using the unique 11-digit personal identity number assigned to all residents of Norway after 1960. Data linkage was performed by a trusted third party (Statistics Norway).

### Study cohort

The study population comprised all people born before 1945 and living in Norway on 1 January 2005. Study subjects were categorized according to birth year; <1915, 1915–1924, 1925–1934, or 1935–1945. Follow-up continued until the first of any censoring events, including the day of any first hip fracture, emigration or death, or until the end of the study period. The study period lasted from 1 January 2005 to 31 December 2010.

### Antihypertensive drugs

The following medications were considered:

- C03A, Thiazides: hydrochlorthiazide, bendroflumethiazide/potassium
- C03C, Loop diuretics: furosemide, bumetanide
- C07, Beta-blockers: propanolol, sotalol, metoprolol, atenolol, bisoprolol, esmolol, labetolol, carvedilol
- C08, Calcium channel blockers: amlodipine, felodipine, isradipine, nifedipine, nimodipine, lercanidipine, verapamil, diltiazem
- C09A, ACE inhibitors: captopril, enalapril, lisinopril, ramipril, trandolapril
- C09B, ACE inhibitor/thiazide: enalapril/thiazide, lisinopril/thiazide, zofenopril/thiazide
- C09C, Angiotensin II receptor blockers: losartan, eprosartan, valsartan, irbesartan, candesartan, telmisartan, olmesartan medoxomil
- C09D, Angiotensin II receptor blockers/thiazide: losartan/thiazide, eprosartan/thiazide, valsartan/thiazide, irbesartan/thiazide, candesartan/thiazide, telmisartan/thiazide, olmesartan medoxomil/thiazide

C02, Antihypertensives (moxonidine, doxazosin, hydralazine, bosentan, ambrisentan) were not included because they were rarely used in “common” patients.

### Drug exposure

The NorPD does not include information on whether or when the dispensed drugs were consumed; hence, we had to make assumptions on drug exposure. The DDD is the assumed average maintenance dose per day for a drug used for its main indication among adults [20]. Since antihypertensive drugs are prescribed primarily for long-term use on a daily basis, the number of days corresponding to the number of DDDs dispensed was used as proxy for the number of person-days exposed. We assumed that people started using the drugs on the day they were dispensed.

We defined overall use as all person-days exposed within the study period (on-treatment definition of exposure). No gap period was included. We defined recently started use as the first 14 days of drug exposure after a 365-day wash-out period.

### Statistical analysis

We compared the incidence of primary hip fracture during the person-days exposed and unexposed to antihypertensive drugs in the study period by calculating the standardized incidence ratio (SIR) [21]. An SIR >1 indicates an increased risk of hip fracture associated with exposure, while an SIR < 1 indicates a decreased risk. We adjusted the SIR for sex, birth year and time period (divided into 2-month intervals).

For SIR values based on fewer than 100 observed primary hip fractures among exposed people, we calculated exact 95 % confidence intervals (CI) assuming a Poisson distribution of the observed number of hip fractures (O) among exposed people, estimating the mean by the expected number of hip fractures among the exposed people. When the observed numbers of hip fractures among exposed people exceeded 100, the 95 % CI values were approximated by the following formula: [SIR · exp (−1.96√O), SIR∙ exp (1.96√O)].

To calculate the attributable effect of exposure to antihypertensive drugs on hip fractures, we divided the observed minus the expected number of fractures during the number of person-days exposed to these drugs by the observed number of fractures in the study population. We performed analysis using IBM SPSS 19 (PASW Statistics for Windows, SPSS Inc, Chicago, IL, USA).

### Ethics and approvals

The Regional Committee for Medical and Health Research Ethics, REC West (138/07) and the Norwegian Data Inspectorate (08/00133) approved the study. We obtained permission from the Norwegian Institute of Public Health regarding data from the Norwegian Prescription Database (08/2173); from the Norwegian Arthroplasty Register regarding data from the Norwegian Hip Fracture Registry (9 June 2009); and from Tax Norway regarding data from the Central Population Registry (2008/612430). The Norwegian Directorate of Health granted an exemption from the duty of confidentiality regarding linkage of data from the three databases (08/1843). Linkage was performed by a trusted third party (Statistics Norway).

## Results

The study cohort comprised 906,422 people (56 % women) with a mean age of 72.8 years (standard deviation (SD) 8.9 years) on 1 January 2005. The distribution by birth year and sex is shown in an additional file [see Additional file 1]. The mean follow-up was 5.2 (SD 1.6) years. During the study period, 218,775 people died (53 % women) and 4949 emigrated (44 % women).

The drugs most frequently used were angiotensin II receptor blockers (30.5 % of the study cohort; plain products: 16.5 %, combination products: 14.0 %), beta-blockers (29.8 %) and calcium channel blockers (21.6 %). More women used diuretics and angiotensin II receptor blockers/thiazide, and fewer women used beta-blockers and ACE inhibitors, as compared with men. Sex differences for other drug groups were minor. The use of antihypertensive drugs decreased with age (Table 1).

**Table 1:** Percentage of people in Norway born before 1945 who purchased antihypertensive drugs during 2005–2010

	Total cohort	By sex		By birth cohort			
	n = 906,422	Women	Men	1935-1944	1925–1934	1915–1924	<1915
		n = 506,568	n = 399, 854	n = 397,761	n = 294,952	n = 183,967	n = 29,742
Thiazide	6.2	7.1	5.2	2.8	2.3	1.0	0.1
Loop diuretic	17.9	19.4	15.9	5.0	7.1	5.3	0.5
Beta blocker	29.8	28.6	31.3	12.1	11.6	5.8	0.3
Calcium channel blocker	21.6	21.5	21.6	8.9	8.4	4.1	0.2
ACE inhibitor	14.0	12.4	16.0	5.4	5.4	2.9	0.3
ACE inhibitor/Thiazide	3.8	2.1	1.7	1.7	1.4	0.7	<0.1
Angiotensin II receptor blocker	16.5	16.8	16.1	7.9	6.0	2.4	0.1
Angiotensin II receptor blocker/Thiazide	14.0	8.1	5.9	7.0	5.1	1.8	0.1

Individuals may have purchased more than one antihypertensive drug type

Altogether, 39,938 (4.4 %) people experienced a primary hip fracture during the study period. On average, 72 % were women; the proportion increased with age, from 63 % (born 1934–1944) to 81 % (born <1915). The mean age at the time of fracture was 83.0 years. People born in 1925–1934 or 1915–1924 experienced most fractures (Table 2).

**Table 2:** Number (n) and incidence rate (%) of hip fracture among people in Norway during 2005–2010

Hip fractures	Birth year							
	1935–1944		1925–1934		1915–1924		<1915	
	n	%	n	%	n	%	n	%
All (n = 39,938)	4904	1.2	13,322	4.5	18,599	10.1	3153	10.6
Women (n = 28,883)	3086	1.5	9269	5.6	13,982	11.8	2546	11.4
Men (n = 11,055)	1818	0.9	4053	3.1	4577	7.0	607	8.3

Table 3 shows that exposure to thiazides (SIR 0.7, 95 % CI 0.6–0.7), beta-blockers (SIR 0.7, 95 % CI 0.7–0.8), calcium channel blockers (SIR 0.8, 95 % CI 0.8–0.8), angiotensin II receptor blockers (SIR 0.8, 95 % CI 0.7–0.8), combination products containing ACE inhibitor/thiazide (SIR 0.7, 95 % CI 0.6–0.7) and angiotensin II receptor blocker/thiazide (SIR 0.6, 95 % CI 0.6–0.6) was associated with decreased risk of hip fracture. Use of loop diuretics and ACE inhibitors was associated with increased risk of fracture if born after 1924, and with decreased risk if born before 1925. The SIRs were lower among exposed men than among exposed women for all drugs except loop diuretics. Generally, the SIRs decreased with increasing age among exposed people, except for thiazides and angiotensin II receptor blockers (Table 3).

**Table 3:** Comparison of number of hip fractures (n) during exposed and unexposed person-time^a^ (SIR, 95 % CI)

	Total cohort		By sex				By birth cohort								Attributable effect (%)
Exposed person-days (DDD)			Women		Men		1935–1944		1925–1934		1915–1924		<1915		
	n	SIR (CI)	n	SIR (CI)	n	SIR (CI)	n	SIR (C)I)	n	SIR (CI)	n	SIR (CI)	n	SIR (CI)	
Thiazide	550	0.7 (0.6–0.7)	441	0.7 (0.6–0.8)	109	0.6 (0.5–0.8)	61	0.7 (0.5–0.8)	208	0.6 (0.6–0.7)	258	0.7 (0.6–0.8)	23	0.8 (0.5–1.2)	–0.6
Loop diuretic	4752	1.0 (1.0–1.0)	3422	1.0 (0.9–1.0)	1330	1.1 (1.1–1.2)	371	2.0 (1.8–2.3)	1538	1.3 (1.2–1.3)	2543	0.9 (0.8–0.9)	300	0.7 (0.6–0.8)	0.1
Beta blocker	4074	0.7 (0.7–0.8)	3010	0.8 (0.8–0.8)	1064	0.7 (0.6–0.7)	511	0.9 (0.9–1.0)	1700	0.8 (0.8–0.8)	1757	0.7 (0.6–0.7)	106	0.6 (0.5–0.8)	–3.5
Calcium channel blocker	5028	0.8 (0.8–0.8)	3736	0.8 (0.8–0.8)	1292	0.7 (0.7–0.8)	642	1.0 (0.9–1.1)	1982	0.8 (0.8–0.8)	2234	0.7 (0.7–0.8)	170	0.7 (0.6–0.8)	–3.4
ACE inhibitor	3438	0.9 (0.9–1.0)	2351	1.0 (0.9–1.0)	1087	0.9 (0.8–0.9)	410	1.3 (1.1–1.4)	1389	1.1 (1.0–1.1)	1512	0.8 (0.8–0.8)	127	0.7 (0.6–0.9)	–0.6
ACE inhibitor/Thiazide	662	0.7 (0.6–0.7)	520	0.7 (0.6–0.8)	142	0.5 (0.4–0.6)	111	1.9 (0.8–1.2)	264	0.7 (0.6–0.7)	261	0.6 (0.5–0.6)	26	0.9 (0.6–1.3)	–0.9
Angiotensin II receptor blocker	2631	0.8 (0.7–0.8)	2041	0.8 (0.8–0.8)	590	0.7 (0.6–0.7)	411	0.9 (0.8–1.0)	1049	0.7 (0.7–0.8)	1094	0.8 (0.7–0.8)	77	0.8 (0.6–1.0)	–1.9
Angiotensin II receptor blocker/Thiazide	2122	0.6 (0.6–0.6)	1688	0.6 (0.6–0.7)	434	0.5 (0.4–0.5)	367	0.7 (0.6–0.7)	915	0.6 (0.6–0.6)	790	0.6 (0.5–0.6)	50	0.6 (0.5–0.9)	–3.6

*SIR* Standardized Incidence Ratio, *DDD* Defined Daily Dose, *Attributable effect* percentage of hip fractures during DDD exposure throughout the study period

^a^The population of Norway born before 1945 and exposed to various antihypertensive drugs in 2005–2010 (exposed person-days, DDD)

Subanalysis for recently started drug treatment revealed increased risk of hip fracture during the first 14 days of treatment with loop diuretics (all: SIR 1.6, 95 % CI 1.3–1.9; women: SIR 1.6, 95 % CI 1.2–2.0; men: SIR 1.6, 95 % CI 1.1–2.3). The number of hip fractures during the first 14 days of treatment was small (*n* = 257) and revealed no statistically significant results for other drug classes (Table 4).

**Table 4:** Recently started antihypertensive drugs; comparison of number of hip fractures during exposed and unexposed person-time^a^

	Total cohort		By sex			
Exposed person-days (14 days)			Women		Men	
	n	SIR (CI)	n	SIR (CI)	n	SIR (CI)
Thiazide	16	0.9 (0.5–1.5)	12	0.9 (0.5–1.6)	4	0.8 (0.2–2.2)
Loop diuretic	104	1.5 (1.3–1.9)	76	1.6 (1.2–2.0)	28	1.5 (1.0–2.1)
Beta blocker	47	1.0 (0.7–1.3)	32	1.0 (0.7–1.3)	15	1.0 (0.6–1.6)
Calcium channel blocker	38	1.0 (0.7–1.4)	30	1.1 (0.7–1.5)	8	0.8 (0.3–1.5)
ACE inhibitor	29	1.0 (0.7–1.5)	24	1.3 (0.8–1.9)	5	0.5 (0.2–1.2)
ACE inhibitor/Thiazide	2	0.4 (0.1–1.6)	2	0.6 (0.1–2.2)	0	–
Angiotensin II receptor blocker	11	1.0 (0.5–1.8)	11	1.0 (0.5–1.8)	0	–
Angiotensin II receptor blocker/Thiazide	10	0.5 (0.2–0.9)	8	0.5 (0.2–1.1)	2	0.4 (0–1.4)

*SIR* Standardized Incidence Ratio

^a^The population of Norway born before 1945 and exposed to various antihypertensive drugs in 2005–2010 after 365 days wash out (exposed person-days, 14 days)

Attributable effect for overall exposure was estimated at −3.6 % for angiotensin II receptor blockers/thiazide, −3.5 % for beta-blockers, and −3.4 % for calcium channel blockers (Table 3).

## Discussion

In this registry-based cohort study including the entire population of Norway aged 60 years and older, we found a reduction in risk of hip fracture associated with use of most antihypertensive drugs. However, fracture risk among users of loop diuretics and plain ACE inhibitors was increased in people younger than 80 years, and in new users of loop diuretics.

### Methodological considerations

The nationwide cohort design is suitable to compare people exposed and non-exposed to antihypertensive drugs with regard to the relatively infrequent outcome, hip fracture, without being prone to selection and recall bias. The health registries provided us a unique opportunity to link complete data on all antihypertensive drugs purchased by a large unselected community-dwelling older population with all primary hip fractures registered in Norway, and the 6-year follow-up period yielded a high number of cases.

However, the databases have some limitations. The NorPD lacks individual information on medications dispensed to people staying in nursing homes (predominantly long-term care) and hospitals (mostly short stays), leading to systematic misclassification as drug non-users. Because frail old people in nursing homes are particularly prone to both treatment with antihypertensive drugs and hip fracture [22, 23], bias from immeasurable exposure time probably caused underestimation of associations among exposed people. The Norwegian Hip Fracture Registry comprised about 90 % of all hip fracture operations in Norway [24], with somewhat lower completeness during the first years. Unfortunately, clinical information regarding diagnoses, BMD, functional level, socioeconomic factors and life style was not available from the included or any other registry. This hampered adjustments for potentially confounding factors such as fall-risk-related comorbidities (FRICs), i.e. heart failure, ischemic heart disease, chronic obstructive lung disease, dementia, depression, Parkinson’s disease and stroke. Many older people treated with antihypertensive drugs probably use other drugs concomitantly, e.g. fall-risk-increasing drugs (FRIDs) such as other cardiovascular drugs, psychotropics, opioids and systemic steroids. However, with the time-varying exposure used in the analysis (SIR), matching exposure periods for other drugs was not possible. We consider the time-varying exposure a major strength of our study, as the alternative, fixed exposure, would have led to extensive and unmeasurable misclassification yielding unreliable results. In Norway, antihypertensive drugs are mainly prescribed for hypertension (88–99 % of overall drug volume); however about 40 % of ACE inhibitors (plain products) and beta-blockers are prescribed for conditions such as heart failure and ischemic heart disease [8], and loop diuretics are mainly used for fluid retention. Because many drugs have several different indications, we decided not to use drugs as proxies of comorbidities. The Norwegian Prescription Database includes reimbursement codes for drugs prescribed for chronic conditions, linked to ICD-10 diagnoses. We did not consider this information because reimbursement codes were neither validated nor complete (selected drugs only) and not available before 2009. Previous studies generally revealed protective associations between hip fracture and antihypertensive drugs to remain when adjusting for comorbidity, socioeconomic factors, life style and concomitant drug use [25, 26]; loop diuretics, however, were sensitive for adjustment [26].

Because antihypertensive drugs are predominantly used on a daily basis, we considered the number of days corresponding to the number of DDDs dispensed the best proxy for the number of drug-exposed person-days. A study in Germany observed the actually prescribed mean daily dose (PDD) to be nearly 2.0 DDD for ACE inhibitors and angiotensin II receptor blockers and close to 1.0 DDD for beta-blockers, calcium-channel blockers and thiazides [27]. Consequently, we may have overestimated the exposed person-time, and thus the strength of the associations between ACE inhibitors and angiotensin II receptor blockers, and the risk of hip fractures.

Health care in Norway is public and tax-financed; very low out-of-pocket money and good reimbursement systems may explain the greater use of newer and more expensive antihypertensive drugs in Norway compared to other countries in Europe and North America [9]. One could suspect that the indication and frequency of treatment with antihypertensive drugs differs in a public health care setting and a setting with insurance based health care.

### Antihypertensive drugs and hip fracture

Different mechanisms have been postulated for effects of antihypertensive drugs on fracture risk depending on the duration of drug use; through hypotension and falls in recently started treatment and through changes in bone metabolism and bone strength in long-term treatment [26]. In addition to direct (falls) and indirect (BMD) drug effects, treatment of the underlying cardiovascular disease may possible reverse its negative effects on bone quality and contribute to reduction in fracture risk.

### Renin-angiotensin-aldosterone active agents

We found the risk of hip fracture to be decreased with angiotensin II receptor blockers (plain and combination products); use of ACE inhibitors was associated with increased risk among users aged <80 years and decreased among those 80 years and older. The human studies conducted previously have provided conflicting evidence regarding the effects of ACE inhibitors on bone metabolism and fracture risk, whereas angiotensin II receptor blockers appear to be osteoprotective. A population-based case–control study in Denmark reported a 14 % reduction in hip fracture risk (adjusted OR 0.86, 95 % CI 0.80-0.92) in users of ACE inhibitor compared with nonusers; the crude OR was not significant. There were no major differences according to sex, age and drug dosage [25]. Another case–control study in the United Kingdom revealed a decreased risk of *any fracture* for longer-term current users (≥20 prescriptions) of ACE inhibitors (adjusted OR, 0.81; 95 % CI 0.73–0.89), though, subanalysis for hip fracture was not conducted. No difference in fracture risk was found with use of angiotensin II receptor blockers [28]. In a large cohort study among low-income older people the United States, new use of angiotensin II receptor blockers was associated with a reduction in hip fracture risk (adjusted Hazard Ratio, HR 0.77, 95 % CI 0.64–0.93) compared with calcium channel blockers (reference exposure), whereas no trend was observed for ACE inhibitors (HR 0.95, 95 % CI 0.84–1.07). Of note, nonusers were not considered, and wash out period (30 days) and follow-up period (median 70 days) were short [26]. In a population-based cohort study in Sweden, use of renin-angiotensin-aldosterone active agents was not associated with altered risk of hip fracture (adjusted OR 0.93, 95 % CI 0.79–1.09); however, separate analyses for ACE inhibitors and angiotensin II receptor blockers were not conducted [29]. Another population-based cohort study in Canada failed to demonstrate significant differences in hip fracture risk in hypertensive older people treated with angiotensin II receptor blockers compared to ACE inhibitors (adjusted HR 0.99, 95 % CI 0.78–1.25); nonusers were not considered [30]. The Hypertension in the Very Elderly Trial (HYVET), a randomized trial with hip fracture as a secondary outcome measure, revealed that treatment with a thiazide-like diuretic ± ACE inhibitor may reduce the risk of hip fracture [31]. To the best of our knowledge, previous research has not included combination products. We found protective associations of ACE inhibitor/thiazide and angiotensin II receptor blocker/thiazide with hip fracture to be stronger than for the individual drugs involved. This may possibly be due to several interacting, beneficial effects on underlying cardiovascular disease and bone strength.

A cross-sectional study of hypertensive Chinese men and women reported associations between ACE inhibitor use and higher BMD at the hip, after adjusting for diagnoses, medications and lifestyle [32]. This finding was not confirmed by a more recent prospective cohort study in the United States; ACE inhibitors were associated with hip bone loss whereas angiotensin II receptor blockers were not [33]. An open randomized trial showed beneficial effects of ACE inhibitors ± thiazide in hypertensive people, i.e. BMD loss was avoided and metabolic profile was improved. Subgroup analysis for ACE polymorphism revealed that beneficial BMD response was confined to women with the DD polymorphism [34]. Another open randomized study demonstrated that angiotensin II receptor blockers inhibited bone loss and increased BMD at the hip in older bed-ridden women, as compared to calcium channel blockers [35]. In vitro, angiotensin I stimulates bone resorption in co-cultures of osteoblast and osteoclasts, and this action is inhibited by ACE inhibitors [36]. Angiotensin II accelerates bone resorption by activating osteoclasts, whereas these effects are blocked by angiotensin II receptor blockers [37]. Hence, animal studies suggest that renin-angiotensin-aldosterone active agents inhibit bone resorption, rather than stimulate bone formation [37, 38].

### Calcium channel blockers

Our finding of a reduced fracture risk among users of calcium channel blockers is partly in line with the above-mentioned epidemiologic studies conducted in Denmark and Sweden. Rejnmark et al. reported a small reduction in hip fracture risk by 7 % (adjusted OR 0.93, 95 % CI 0.87–0.99) [25] while Thorell et al. found a reduction by 18 % (crude OR 0.82, 95 % CI 0.68–0.98); the differences in the latter study were not maintained, however, after adjustment for age, sex and comorbidity [29]. Although in vitro studies suggest that calcium-channel blockers may inhibit osteoclast function [38], previous research has found no association with hip BMD [32].

### Thiazides

Our findings of a reduced hip fracture risk among thiazide-users align with a Cochrane review including 6 cohort and 15 case–control studies; compared with nonusers, the fracture risk among thiazide users was decreased by 24 %, relative risk (RR) 0.76 (95 % CI 0.64–0.78) [39]. Randomized controlled trials have revealed preservation of cortical bone in older women and men using thiazides, however, fracture was not an outcome measure [40, 41]. Beneficial effects on BMD are probably caused by increased renal calcium absorption, suppressed parathyroid hormone secretion and enhanced calcium uptake in the intestine, leading to a positive calcium balance. Further, thiazides directly affect bone by stimulating osteoblast differentiation [15].

### Loop diuretics

The results of our study suggest that risk of hip fracture with loop diuretics changes with the users’ age; fracture risk being increased among users aged <80 years, and decreased among those 80 years and older. A meta-analysis of 4 cohort and 9 case–control studies reported a 14 % increase in overall risk of hip fracture among loop diuretic users compared with nonusers, RR 1.14 (95 % CI 1.08–1.19) [42]. However, most included studies were adjusted for age, not allowing for comparison between different age groups. Some studies have reported sensitivity of risk estimates to adjustment for other confounders (e.g. prior fracture, comorbidity or use of other drugs), revealing smaller RRs after adjustment [26, 43]. Cohort studies and a randomized controlled trial have reported loss of total hip BMD among older women and men using loop diuretics compared with nonusers [44–46]. Interestingly, a recent study indicated a clinical association of hyponatremia during loop diuretic use and an increased risk of osteoporotic fractures, possible due to exertion of loss of bone sodium and calcium [47].

### Beta-blockers

We found the risk of hip fracture to be decreased with use of beta-blockers, in accordance with two meta-analyses. Yang et al. (6 cohort and 7 case–control studies) reported reduction in risk by 17 % (RR 0.83, 95 % CI 0.70–0.92) [48], and Toulis et al. (7 cohort and 9 case–control studies) reduction by 14 % in women (pooled effect size (ES) 0.86, 95 % CI 0.80–0.91) and 20 % in men (ES 0.80, 95 % CI 0.71–0.90) [49]. Although observational studies have associated use of beta-blockers with a higher BMD at the hip [50–52], randomized trials are lacking. Several studies suggest the reduction in hip fracture risk to be associated with β1-selective agents rather than nonselective beta-blockers [49, 53]. The underlying mechanisms are not yet clear. Increased sympathetic nervous activity causes an increase in bone resorption and a decrease in bone formation through stimulation of osteoclasts and inhibition of osteoblasts. In vitro studies suggest that beta-blockers enhance bone formation through blocking input from the sympathetic nervous system [54].

### Recently started drug use

We found increased risk of hip fracture during the first 2 weeks of treatment with loop diuretics; similar findings have been reported by Berry et al. for the initial 3 weeks of treatment with thiazides and loop diuretics [12], and by Butt et al. for the 45 days immediate after a first prescription of various antihypertensive drugs (incidence rate ratio 1.43, 95 % CI 1.19–1.72) [11]. Although the numbers of hip fractures in these studies were relatively small and the findings must be interpreted with caution, the results are interesting because they are opposite to those with long-term drug use. Whereas initiating antihypertensive drugs is known as risk factor for falls in older people [11], two meta-analyses revealed no significant associations of falls with current use of antihypertensive drugs [13, 14]. On the other hand, a recent population-based study found a dose–response relationship between drug dose and falls [55].

### Age and sex

We found a lower risk of hip fracture among exposed men than among exposed women. Rejnmark et al. reported similar trends for sex with regard to beta-blockers, ACE inhibitors and calcium channel blockers [25]. Sex hormones interfere with bone metabolism, posing postmenopausal women at a much higher risk of hip fracture than men; most antihypertensive drugs appear to be beneficial for bone health in both sexes, however, risk differences are not eliminated.

Generally, we found the protective associations to be most evident within the youngest cohorts. We probably underestimated the risk of hip fracture among the oldest old because the lack of clinical information (confounding factors could not be adjusted for) and the systematic misclassification of the highly exposed [22] nursing home patients as drug non-users. Amplification of the effects of these drugs with advancing age may suggest the maintenance of protective drug effects despite other risk factors such as frailty, osteoporosis, comorbidity and concomitant drug use. On the other hand, cessation of drug treatment in the oldest age groups could be associated with general clinical deterioration (the “healthy user effect”), explaining the SIRs below 1. The increased hip fracture risk in people younger than 80 years using loop diuretics and plain ACE inhibitors, however, may possibly be due to the “more-ill-user effect” as these drugs are frequently issued to relatively frail people with heart failure.

## Conclusions

Our findings support that older people using antihypertensive drugs are at a lower risk of hip fracture compared with people not using these drugs. Fracture risk among users of loop diuretics and ACE inhibitors younger than 80 years, however, was increased. This may have great impact at the population level, because the use of antihypertensive drugs is widespread in people at risk of hip fracture. Clinical studies are needed to further explore these associations. Even though antihypertensive drugs might be protective against hip fracture, prescribers should carefully consider increased vulnerability to hemodynamic side effects when initiating treatment in older people and practice the key principle: “start low and go slow.”

## Additional file

Additional file 1:
**Number of people in Norway born before 1945 by birth cohort and sex distribution.** (DOC 29 kb)
