# Supplementary material for: Risk of hip fracture among older people using antihypertensive drugs: a nationwide cohort study
Source: BMC Geriatr. 2015 Dec 1;15:153. doi: 10.1186/s12877-015-0154-5 (PMC4667446; doi:10.1186/s12877-015-0154-5)
Supplement: Additional file 1: — Number of people in Norway born before 1945 by birth cohort and sex distribution. (DOC 29 kb) [file 12877_2015_154_MOESM1_ESM.doc]

Additional file 1 Number of people in Norway born before 1945 by birth cohort and sex distribution

|  | **Birth year** | | | |
| --- | --- | --- | --- | --- |
| **Study cohort** | **1935–1944** | **1925–1934** | **1915–1924** | **<1915** |
| **All (n = 906,422)** | 397,761 | 294,952 | 183,967 | 29,742 |
| **Women (n = 506,568)** | 202,399 | 163,240 | 118,519 | 22,410 |
| **Men (n = 399,854)** | 195,362 | 131,712 | 65,448 | 7,332 |

Distribution by January 1^st^ 2010
